# State-of-the-art for CAR T-cell therapy for chronic lymphocytic leukemia in 2019

**DOI:** 10.1186/s40425-019-0686-x

**Published:** 2019-08-01

**Authors:** Richard Lemal, Olivier Tournilhac

**Affiliations:** 1 Histocompatibility laboratory, CHU Clermont-Ferrand, INSERM CIC501, EA 7453 -Université Clermont Auvergne, Clermont-Ferrand, France; 2 Adult Clinical Hematology, CHU Clermont-Ferrand, INSERM CIC501, EA 7453 -Université Clermont Auvergne, Clermont-Ferrand, France

**Keywords:** CLL, CAR T cells, Ibrutinib

## Abstract

Experience in the use of CAR T cells to treat CLL is limited, but safety and efficacy data are encouraging, suggesting that it may be possible to use CAR T cells in populations of CLL patients with particularly unfavorable prognoses. Mechanisms intrinsic to the pathophysiology of CLL undoubtedly explain the efficacy reported based on limited data for the first few series, and underlie the rationale of successive modulations in lymphodepletion schemes, transgene constructs, and, finally, the therapeutic association of CAR T cells with ibrutinib, which appears to be particularly promising. This review describes the published results and expected developments.

## Introduction

Chronic lymphocytic leukemia (CLL) is the most common lymphoid hemopathy (estimated incidence of 2 to 4 cases per 100,000 inhabitants/year). It is diagnosed at a median age of 72 years, and therefore mostly in patients with comorbid conditions [1]. It is a B lymphoid hemopathy characterized by invasion of the bone marrow, blood, and secondary lymphoid organs (spleen and/or lymph nodes). Prognosis is evaluated essentially on the basis of cytogenetic and molecular biology analyses. The two most unfavorable elements associated with a poor prognosis are: 1) p53 alterations (17p deletion and/or *TP53* gene mutation), which weaken the response to cytotoxic agents, and 2) a complex karyotype (with more than three abnormalities) [1]. Treatment indications are based on the progressivity criteria of the International Workshop on CLL (IWCLL) [2]. The recent development of BCR pathway inhibitors (BCRi; Bruton Tyrosine Kinase (BTK) and PI3Kδ inhibitors) and BCL2 inhibitors (BCL2i) has completely modified the therapeutic landscape of CLL [3], but the extent of these changes remains unclear [4]. However, patients with relapses or with tumors refractory to such treatments still have an unfavorable prognosis. Hematopoietic stem cell allografts (generally followed by monitoring and preemptive treatment of residual disease [5]) remain a possible treatment, but its use is clearly declining [6] and is generally reserved for rare eligible CLL patients with a very poor prognosis.

T cells bearing a chimeric antigen receptor (CAR T cells) are generated by genetic engineering, and are designed to arm the immunocompetent T cells of the patient with an activating receptor consisting of 1) an extracytoplasmic variable fragment of an immunoglobulin (scFv) directed against a tumor target, 2) an intracellular T-cell receptor activation molecule (CD3ζ) and 3) positive costimulation molecules (generally CD28 and/or 4-1BB) [7]. The result is a population of immune cells, mostly T lymphocytes, capable of recognizing a tumor target without Major Histocompatibility Complex (MHC) restriction, and destroying that target through cytotoxic effector mechanisms. The most advanced CAR T cells developed to date are directed against CD19: tisagenlecleucel and axicabtagene ciloleucel, both released onto the market in the United States and Europe in 2017/2018, tisagenlecleucel for use against pediatric B acute lymphoblastic leukemia (B-ALL) and diffuse large B-cell lymphoma (DLBCL) in relapse or refractory to standard immunochemotherapy treatment and axicabtagene ciloleucel for DLBCL only [7].

CLL was one of the first diseases in which CAR T cells were used [8], but experience with the use of this treatment is currently less extensive for this disease than for B-ALL or DLBCL. The objective of this review is to discuss the main results obtained with CAR T cells in CLL and to consider likely developments.

### Efficacy data

Since the first report of the efficacy of second-generation CAR T cells against CLL in 2011 [8], results have been published or reported for the injection of CAR T cells into 134 CLL patients [8–22]. The clinical status of these patients is reported in Table 1, together with the CAR T constructs and lymphodepletion schemes used.

**Table 1:** Clinical situations and the characteristics of the CAR T cells used to treat the 134 CLL patients reported to date

	Clinical context						CAR T characteristics					Efficacy	Toxicity		
Reference/year	Number of CLL patients	Clinical situation	Prior ttmt with ibrutinib	Prior ttmt with venetoclax	TP53 alterations	Complex karyotype	Targeted Ag	Co-stimulation	Cell source	Lymphodepletion	Treatment combination	Responses	CRS all grades *N* (%)	CRES all grades *N* (%)	Toxcity-related mortality
[8] / 2011	1	R/R	0	0	1/1	0	CD19	4-1BB	Autologous	P + C	None	CR	1 (100)	0	0
[9] / 2011	8	R/R	0	0	2/8	1/8	CD19	CD28	Autologous	None or C	None	3/8 SD	8 (100)	0	1(13)
[10] / 2011	3	R/R	0	0	2/3	0	CD19	4-1BB	Autologous	B + R or P + C	None	3/3 ORR 2/3 CR	3 (100)	0	0
[11] / 2012	4	R/R	0	0	ND	ND	CD19	CD28	Autologous	F + C	IL2 IV for 5 d	3/4 ORR 1 CR	4 (100)	0	0
[12] / 2013	4 2 Richter	Recurrence after allogeneic treatment	0	0	2/4	ND	CD19	CD28	Allogeneic	None	None	1/4 ORR 1 PR 1 SD	ND	ND	0
[13] / 2015	5 1 Richter	R/R	ND	ND	ND	ND	CD19	CD28	Autologous	F + C	None	5/5 ORR 3 CR 2 PR	3 (60)	1 (20)	0
[14] / 2015	14	R/R	1/14	0	6/14	ND	CD19	4-1BB	Autologous	B or P+ C or F + C	None	8/14 ORR (57%) 4/14 CR (28%) 4/14 PR (28%) 4 MRD neg	9 (64)	5 (36)	0
[15] / 2016	3	R/R	3/3	0	3/3	2/3	CD19	4-1BB	Autologous	ND	Ibrutinib stopped just before leukapheresis	3/3 ORR 1 CR 2 PR	ND	ND	ND
[16] / 2016	5	Recurrence after allogeneic treatment	ND	ND	ND	ND	CD19	CD28	Allogeneic	None	None	2/5 ORR 1 CR 1 PR	4 (80)	0	0
[17] / 2016	2	R/R	0	0	ND	ND	Kappa	CD28	Autologous	None	None	0 ORR 1 SD	ND	ND	ND
[18] / 2017	24 5 Richter	R/R post-ibrutinib 25% post venetoclax	24/24	6/24	23/24	16/24	CD19	4-1BB	Autologous	F + C mostly, or F or C	None	71% ORR 21% CR 43% PR 58% MRD neg	20 (83)	8 (33)	1 (4)
[19] / 2018	8	1st line P + FC	0	0	0	0	CD19	CD28	Autologous	C	None	3/8 ORR (38%) 2/8 CR	4 (50)	0	0
[20] / 2018	19 4 Richter	R/R post-ibrutinib	19/19	11/19	14/19	14/19	CD19	4-1BB	Autologous	F + C	Concomitant ibrutinib	15/18 ORR (83%) 11/13 CR BM with MRD neg	14 (74)	6 (32)	1 (5)
[21] / 2018	19	R/R ×14 +1st line ibrutinib ×5	5 in 1st line	0	11/19	ND	CD19	4-1BB	Autologous	ND“chemotherapy”	Concomitant ibrutinib	10/11 ORR 94 CR BM with 78% MRD neg	18 (95)	5 (26)	0
[22] / 2018	16	R/R post-ibrutinib	16/16	8/16	10/16	8/16	CD19	4-1BB	Autologous	F + C	None	81.3% ORR 7 CR 6 PR	12 (75)	1 (6)	0

The patient from reference [8] is also reported in reference [10] and the 3 patients from the reference [10] are also reported in reference [14]. One patient reported in reference [11] is also reported in reference [13]

*R/R* relapse/refractory disease, *ORR* overall response rate, *CR* complete response, *PR* partial response, *SD* stable disease, *ND* no data, *Ttmt* treatment, *IV* intravenous, *C* cyclophosphamide, *B* bendamustine, *R* rituximab, *P* pentostatin, *F* fludarabine, *CRS* cytokine release syndrome, *CRES* CAR T cell-related encephalopathy syndrome

The first observation to emerge from these results is that the population of treated patients had a particularly poor prognosis. The median age of the patients treated was 61 years (range: 40 to 77 years), and most were in relapse after a large number of lines of treatment. Overall, 68 patients had already received ibrutinib [14, 15, 18, 20–22], 25 had already received venetoclax [18, 20, 22], nine were in post-allograft relapse [12, 16], and 12 were treated in the context of transformation into refractory high-grade lymphoma (Richter’s syndrome) [12, 13, 18, 20]. In addition, 74 of the 108 patients evaluated (68.5%) had p53 alterations, and 41 of the 70 patients evaluated (58.6%) had a complex karyotype (see Table 1).

It is not straightforward to integrate these data, but a second observation that emerges is that efficacy is lower for CLL than for B-ALL and DLBCL: a complete response (CR), according to the IWCLL criteria, was obtained in only a minority (20–30%) of patients [14, 18], and progression-free survival (PFS) estimated at 25% at 18 months [14, 23]. Responses appear to be weaker in the lymph nodes than in the bone marrow and blood. Furthermore, these results should be considered in light of the frequency of complete bone marrow responses with undetectable minimal residual disease (MRD) reported in some series [18, 20–22], which has been correlated with PFS and OS close to 100%, with a median follow-up of 6.6 months [18]. It is difficult to determine the precise response to CAR T cells specifically in patients with Richter’s syndrome from published data, but this response is objective, with a possible decrease in lymph-node tumor syndrome. However, it appears to be partial and transient, and insufficient for the moment to improve the very poor prognosis of these patients [12, 13, 18, 20].

Promising data have also been obtained for the use of allogeneic CAR T cells derived from lymphocytes from hematopoietic stem cell donors in the context of post-allograft relapse [12, 16]. Response rates remain low in these patients with a poor prognosis, but there are signs of efficacy, and the absence of graft-versus-host disease (GVHd) is highly reassuring.

### Past and future improvements

As for other CAR T cell indications, there have been many improvements in lymphodepletion schemes and the construction of chimeric receptors.

Lymphodepletion was initially achieved with cyclophosphamide treatment alone, but today, it is almost always achieved with a combination of cyclophosphamide and fludarabine (see Table 1). This lymphodepletion procedure makes it possible, in particular, to improve the expansion and persistence of CAR T cells via hypothetical mechanisms such as the decrease in residual tumor mass, the induction of inflammation, the release of tumor antigens, and the decrease in the number of regulatory cells. The immunodepression induced by such lymphodepletion may also decrease the risk of immunization against the extracytoplasmic immunoglobulin variable fragment of CAR T cells, which is mostly of murine origin.

Alternatives to the currently preferred antigenic target in B lymphoid hemopathies, CD19, exist and may prove to be more effective or safe. For example, the use of clonal anti-light chain (kappa or lambda) CAR T cells would, theoretically, spare half the B-cell compartment and limit agammaglobulinemia [17]. CD23, the receptor of the invariant fragment of IgM (FcγR), or ROR1 (tyrosine kinase-like orphan receptor 1) are also potentially interesting targets, as they are relatively specific to the B-cell tumoral compartment of CLL [24–26].

The use of CAR T cells combining the variable fragment and the CD3ζ chain with a co-stimulatory molecule of CD137 (or 4-1BB), rather than CD28, which was used in the first trials [8, 10, 14, 21], or in association with CD28 [18, 20, 22] made it possible to optimize the anti-leukemic effect of CAR T cells and to improve their long-term expansion and persistence via mechanisms that are still only partially understood [27].

The use of a variable fragment of humanized immunoglobulin in the construction of CAR T cells [21] should make it possible to limit the risk of immunization against the variable fragment, as most of the fragments used originate from mice, thereby improving the long-term maintenance of the CAR T population. Control over the CD4/CD8 ratio of the injected CAR T cells [22] could also improve the management of the CAR T cell expansion and long-term maintenance phases.

Finally, it is clear that disease persistence at time on injection has an impact on the expansion and maintenance of CAR T cells, and the composition of the expanding population: indeed, the CAR T cells of CLL patients displaying a CR at the time of injection expand more effectively and have a cytokine profile favoring their cytotoxic function and better long-term maintenance [23, 28, 29]. In addition, toxicity is lower when the residual tumor mass is limited at the time of CAR T-cell injection. These findings argue for administration earlier in the course of the disease, to ensure that the best possible response is obtained.

### Immunosubversion in CLL: an obstacle for CAR T cells

The lower efficacy of CAR T cells in CLL may be partly due to the intrinsic characteristics of the immune system in CLL, which is exhausted by diverse immunosubversion mechanisms, decreasing CAR T-cell activation after transduction.

Indeed, the CD4+ T cells of CLL patients have an exhausted phenotype (strong expression of PD-1, CD160, and CD244) and their CD8+ T cells have low proliferative and cytotoxic capacities [30]. These intrinsic characteristics of CLL immune cells are present at the time of diagnosis, but are also favored by previous lines of treatment (with fludarabine, in particular).

The ex vivo expansion and transduction capacities of T cells from CLL patients are clearly different from those of T cells from healthy subjects. In particular, T cells from CLL patients display less expansion of so-called “naïve” CD4+ T cells, an essential criterion for the long-term activity of CAR T cells. Moreover, the naïve CD4+ T cells that manage to expand from the autologous samples of CLL patients express more exhaustion markers [28].

These data support a rationale of developing allogeneic CAR T cells from a healthy donor, in whom the capacity of T cells to expand and their cytotoxicity are not modified by the tumor clone.

### Ibrutinib for CAR T cell optimization?

Ibrutinib has already revolutionized the routine management of CLL, but it may also improve outcomes in CLL patients receiving CAR T cells.

Indeed, particularly promising rates of response to CAR T therapy were reported in three studies. In 2016, Fraietta et al. reported their experience with this treatment, which was limited to three patients who stopped taking ibrutinib just before the leukapheresis preceding CAR T therapy. A response was observed in all three patients, including complete remission in one case, despite the absence of lymphodepletion [15]. At the last American Society of Hematology conference, two groups reported results for two series of 19 patients receiving injections of structurally different CAR T cells, in combination with ibrutinib. The overall response rate was above 80% and the frequency of complete bone-marrow response with undetectable MRD exceeded 90% [20, 21].

Many hypotheses have been put forward to explain this effect of ibrutinib, mostly based on our knowledge of the impact of ibrutinib on the immune system in CLL, which is probably still very patchy. In addition to Bruton’s tyrosine kinase, ibrutinib is known to target the IL2-inducible T-cell kinase (ITK), which orients T cells towards a Th1 cytokine secretion profile [31]. Ibrutinib may therefore be involved in redirecting the immune response of autologous T cells (before and after transduction) from a Th2 profile to a Th1 profile, more favorable for the long-term expansion and maintenance of chimeric receptor-expressing T-cell populations. Indeed, the ability of ibrutinib to promote the expansion, maintenance, and cytotoxicity of CAR T cells and to promote cellular immune responses (with, in particular, a decrease in exhaustion markers, the modification of cytokine secretion profiles, and an increase in the diversity of the T repertoire, etc.) has been demonstrated in vitro [15, 32, 33].

### Safety data

Cytokine release syndrome (CRS) and neurological toxicity (CRES, for CAR T cell-related encephalopathy syndrome) are, as in other indications for CAR T therapy, the most frequent complications in CLL, and their management is not different in this context [7, 34]. The incidence of these complications is variable in the small series available and it is probably still difficult to compare them: CRS occurs in 50 to 100% of patients (Grade ≥ 3 in 25 to 60% of cases), whereas neurological toxicity is less common (0 to 35% of cases) and mostly of moderate intensity. Death attributable to the CAR T cell procedure was reported for three of the 129 patients for whom clinical outcome data are available (2.3%).

CAR T cells do not appear to behave differently in CLL and in other hematological diseases in terms of the time lag to the onset of complications or the response to tocilizumab or corticosteroids, and there are, therefore, currently no specific instructions for CLL.

The use of ibrutinib before leukapheresis has been linked to a higher incidence and greater severity of CRS in first series [18], but the concomitant administration of ibrutinib and CAR T cells appears to be associated with a lower incidence of ≥ grade 3 CRS [20, 21] and lower levels of pro-inflammatory cytokines (including IL-6, IL2Rα, and MCP-1, in particular) [20].

Finally, in one case, a patient with CLL treated with CAR T cells was reported to display the proliferation of an identified population of clonal CD8+ CAR T cells carrying 1) a *TET2* gene interrupted by the chimeric antigen receptor transgene and 2) a preexisting *TET2* mutation in the second allele [35]. This resulted, in this particular case, in the persistence of the mutated *TET2* CD8+ CAR T-cell population and complete remission of CLL more than five years after injection. This example, presented as an opportunity by the authors, should make us think carefully about the relatively moderate control we have over such genetic manipulations, particularly in elderly patients who have received a number of different treatments, in whom residual hematopoiesis is fragile and oligoclonal, and about the need to follow these patients closely in the long term.

### Future changes in indications

Therapeutic strategies for CLL will be dominated, in the near future, by the use of BCRi, which will be the first-line treatment in most patients, relegating immunochemotherapy to an uncertain secondary role. BCL2i is currently indicated for patients with relapses and patients intolerant to BCRi. This new strategy certainly has major advantages in terms of response and survival, but several obstacles to its use have emerged: 1) the use of these new drugs, often continuously until relapse is associated with adverse effects, including cardiovascular effects (for BCRi) and with very high direct costs. CAR T cells could be used early in the treatment of CLL, as an alternative. 2) The treatment of patients with relapses or refractory disease after treatment with BCRi and BCL2i and the treatment of patients with Richter’s syndrome remain challenging. In these high-risk patients, CAR T cells are currently used 1) in place of HSC allografts for patients ineligible for HSC transplantation and 2) instead of HSC allografts for some patients eligible for transplantation. However, CAR T cells could ultimately be used as a complementary treatment, in addition to HSC allografts.

## Conclusion

The CLL treatment paradigm has been deeply modified by the availability of new treatments including BCRi and BCL2i, allowing patients with relapsed CLL at high risk to benefit from prolonged remission periods. However, relapses remain the rule, especially in patients with adverse biological criteria such as p53 alteration, and complex karyotypes. In patients failing BCRi or BCL2i, CAR T therapy offers a new opportunity that could not only replace allogeneic HCT in patients who would have been eligible, but could also be extended to older patients with a reasonable level of co-morbidity. CAR T therapy could also directly compete with targeted therapies, which because of their mechanism of action, must be administered over the long term, leading to problems of toxicity, compliance and ultimately cost.

CAR T therapy does not solve all the therapeutic challenge in CLL and comes with limiting toxicity in a population whose median age exceeds 70 years and which may have acquired hematopoietic alterations, whose the frequency increases with age.

The optimization of CAR T constructions is a way of improvement. But from now on, the question arises of improving the results based on the CAR-T available in practice and in particular their combination with other CLL therapies. Ibrutinib in this context has been evaluated and its maintenance at the time of injection of CARis a promising option that will be evaluated prospectively (NCT03331198). Beyond BCRi, the place of venetoclax also remains to be defined in this specific context.

## Data Availability

Not applicable

## Abbreviations

B-ALL: B-cell acute lymphoblastic leukemia
BCL2: B-cell lymphoma 2
BCR: B-cell receptor
BTK: Bruton Tyrosine Kinase
CAR T cells: T-cell with chimeric antigen receptor
CLL: Chronic lymphocytic leukemia
CRES: CAR T cells related encephalopathy syndrome
CRS: Cytokine release syndrome
DLBCL: Diffuse large B-cell lymphoma
GVHd: Graft versus host disease
HSC: Hematopoietic stem cell
ITK: IL2-inductible tyrosine kinase
IWCLL: International workshop on CLL
MRD: Minimal residual disease
OS: Overall survival
PFS: Progression-free survival
PI3Kδ: Phospho-inositol 3 kinase delta
